# Marine Robotics for Deep-Sea Specimen Collection: A Systematic Review of Underwater Grippers

**DOI:** 10.3390/s22020648

**Published:** 2022-01-14

**Authors:** Angela Mazzeo, Jacopo Aguzzi, Marcello Calisti, Simonepietro Canese, Fabrizio Vecchi, Sergio Stefanni, Marco Controzzi

**Affiliations:** 1 The BioRobotics Institute, Scuola Superiore Sant’Anna, 56127 Pisa, Italy; marco.controzzi@santannapisa.it; 2 Department of Excellence in Robotics & AI, Scuola Superiore Sant’Anna, 56127 Pisa, Italy; 3 Stazione Zoologica Anton Dohrn (SZN), 80121 Napoli, Italy; jaguzzi@icm.csic.es (J.A.); simonepietro.canese@szn.it (S.C.); fabrizio.vecchi@szn.it (F.V.); sergio.stefanni@szn.it (S.S.); 4 Instituto de Ciencias del Mar (ICM)—Consejo Superior de Investigaciones Científicas (CSIC), 08003 Barcelona, Spain; 5 Lincoln Institute for Agri-Food Technology, University of Lincoln, Lincoln LN6 7TS, UK; mcalisti@lincoln.ac.uk

**Keywords:** underwater gripper, ROV gripper, underwater manipulation, underwater end-effector, robotic underwater hands, marine biological sampling

## Abstract

The collection of delicate deep-sea specimens of biological interest with remotely operated vehicle (ROV) industrial grippers and tools is a long and expensive procedure. Industrial grippers were originally designed for heavy manipulation tasks, while sampling specimens requires dexterity and precision. We describe the grippers and tools commonly used in underwater sampling for scientific purposes, systematically review the state of the art of research in underwater gripping technologies, and identify design trends. We discuss the possibility of executing typical manipulations of sampling procedures with commonly used grippers and research prototypes. Our results indicate that commonly used grippers ensure that the basic actions either of gripping or caging are possible, and their functionality is extended by holding proper tools. Moreover, the approach of the research status seems to have changed its focus in recent years: from the demonstration of the validity of a specific technology (actuation, transmission, sensing) for marine applications, to the solution of specific needs of underwater manipulation. Finally, we summarize the environmental and operational requirements that should be considered in the design of an underwater gripper.

## 1. Introduction

### 1.1. Field-Specific Requirements for Design and Control of Manipulation Tools

Requirements associated with robotic manipulation vary according to the field of operation [1]. Industry, for instance, is the place where robotic manipulation has long since established [2]. Major constraints on the development of robotic systems for industrial settings are related to requirements dictated by the task. Thus, competitiveness among stakeholders has contributed to the validation of efficient systems for pick and place, as well as task-specific tools. The advent of collaborative robotics has changed the requirements for industrial robots, first of all in terms of safety. Collaborative robotics opened new horizons on both technical (adaptive control strategies and multi-modal sensory technologies [3]) and behavioral sides (interdependency between human and robot [4]).

Collaborative interaction is a prerequisite in prostheses design. In this case, the requirements guide affordable, lightweight, limited volume, and energy-efficient devices [5]. The inclusion of multiple functionalities should reach a compromise with ease of control [6]. Social issues are relevant in the prosthetic field, adding the strong requirement of a pleasant cosmetic appearance. Sensors intended for sensory feedback restoration are generally integrated [5].

In space, manipulation systems might substitute astronauts in performing time-consuming actions, or risky tasks, such as extra-vehicular activities [1]. Strict requirements are imposed by the environment and the compatibility to the launching phase, such as lightweight systems that withstand different temperatures. Those environmental requirements add to task-related ones, such as versatility and reconfigurability.

Unmanned underwater vehicles (UUVs) are generally equipped with one or more manipulators, which allow for interaction with the environment. Presently, marine robotic mobility functionalities are prioritized over object manipulation, with apparently less research done in this field, in comparison to biomimetic locomotion designs and energy provision [7]. Typical tasks are offshore industry operations, archeological campaigns, or sampling specimens to perform biological/geological/ecological analyses. A thorough review of commercially available underwater manipulator is available in [8]: although a great number of technological solutions are available, the operation of the manipulator still represents a consistent part of the workload on the pilot [9]. As reported by Galloway et al. [10], most of the commercially available manipulators are designed keeping in mind heavy tasks as pipeline inspection and maintenance. Despite those manipulators not being intended for the collection of delicate structures such as deep-sea biological or archeological samples, the industrial grippers of the manipulators of remotely operated vehicles (ROVs) are nowadays widely employed for scientific sample collection.

### 1.2. Rationales and Objectives of the Proposed Review of Underwater Grippers

Dexterity in robotic manipulation might be achieved with advances in hardware and software [2]. We decided to focus on the field of underwater manipulation for underwater biological sample collection, because the existing grippers can be improved in both design and control strategies. Ensuring the correct completion of the collection procedure is important not only to obtain an intact sample, as required for taxonomical analysis, but also to deliver a specimen (or part of it) which presents a physiological condition as similar as possible to the one before sampling. The preservation of physiological conditions is needed because any alteration may affect the post-sampling analyses.

A more targeted hardware solution, or ideally a solution that is versatile to different applications, may achieve better performances in the collection and manipulation of fragile samples than industrial grippers. On the other side, partial automation of the sampling procedures could relieve the operator from demanding tasks. Obviously, other factors impact the success of sampling procedures in the marine environment, such as turbidity, which might alter vision, or water currents, which might impair manipulation stability.

Regardless of the specific field of application, the design of an effective tool or control strategy starts from a clear understanding of the problem to solve and the solutions currently available [11]. This knowledge is then thoroughly translated into a set of requirements, which sets the ground for the technical description of what needs to be designed. This approach seems to be rarely adopted in underwater grippers development, possibly due to the scarce availability of literature that analyzes underwater manipulation procedures. Similarly, high-level control strategies are difficult to implement if there is little knowledge of the reasoning behind the operator’s choices. To the best of our knowledge, Clark et al. [12] authored one of the few essays on deep-sea biological sample collection. In this book, one chapter is entirely dedicated to sampling with ROV. As concerns literature describing technological solutions, recently, some reviews have focused on soft grippers or manipulators for underwater manipulation [13,14].

Here, we aim to fill this gap in the literature by describing sampling procedures with a series of two papers. This systematic review explores the research status of prototypical solutions for delicate specimen collection underwater. This search is performed considering rigid, soft and mixed solutions comprehensively, and it is meant to point out features of the design that have significantly improved performances in underwater sampling. This work of literature revision lays the ground for the second research paper [15], where we discuss the results of an investigation about sampling procedures, which is carried on by directly asking researchers and ROV pilots to report on their experience. This investigation is needed to frame sampling procedures into a structure which highlights all the possible choices in the sequences of actions. This is useful both to partially automate some of the processes, and to define the task-related requirements for underwater grippers. In [15], at the end, we merge the requirements resulting from the literature review and the investigation from experts into a final comprehensive list, which includes environmental, operational and task-related requirements for a gripper for underwater sampling.

This paper is therefore organized as follows. In Section 2, we describe the procedures adopted to perform the systematic review of the literature. In Section 3, an overview of the sampling tools currently used and the research status of technologies for marine sampling is given. In Section 4, we discuss whether and how the key needs in underwater sampling procedures are satisfied by the available technology, and finally, in Section 5, conclusions are drawn.

## 2. Materials and Methods

In Section 2.1, we present the methodology that we followed to systematically review the scientific literature about underwater grippers, adopting the “preferred reporting items for systematic reviews and meta-analyses” (PRISMA) guidelines [16]. In Section 2.2, we explain the methodology for data extraction and organization.

### 2.1. Studies Selection Process and Eligibility Criteria

The bibliography was retrieved from searching the Scopus database, merging the results from the two query strings shown in Table 1. The search considered conference papers, articles, book chapters and reviews published since 1996 in English. The search results were saved on 7 February 2021.

As a first screening process, titles and abstracts were read, and records were selected according to the inclusion or exclusion criteria listed in Table 2. Then, selected articles were retrieved for full-text reading: at this stage, we selected the papers describing, with sufficient detail, the design, realization and testing of grippers for underwater manipulation. We did not account for concepts of design that were never manufactured, or that did not take into account the design issues typical of the marine environment. Moreover, among series of articles referring to the same gripper technology, we reduced the number of the papers selected, in order to eliminate the redundancy in the extracted information.

During the data extraction phase (see Section 2.2), in the case of disambiguation on the information retrieved, or to fill missing information concerning a gripping technology, further publications mentioning the gripper technology were searched for. Those publications might not have been found in our previous systematic search, because their main topic was not the gripper technology itself. However, in a few cases, they proved necessary to complement all data related to a specific gripping technology, hence, they were added to the list.

### 2.2. Data Extraction

From the publications related to the selected underwater gripping technologies, we extracted the following features: year of publication of the scientific article; technology among soft, rigid or mixed; number of fingers, joints and actuators; weight and dimensions; finger movement ranges and force/torque/speed; type of actuation, transmissions and sensors; sealing measures; target objects or aim; depth of testing and tested objects. Such features were selected by authors’ educated opinions to represent scientific/technological maturity of the prototype and, during the analysis, we also took note of the environmental or operational requirements mentioned throughout the text.

Different criteria could have been used to group the research grippers, as the innovation introduced (technological or task-related) or the type of actuation used (hydraulic, electric, pneumatic). For the purpose of this paper, which is the definition of design requirements for a gripper for sample collection in the deep-sea, we classified the research status of the grippers according to the testing environment. This classification does not consider the testing environment reported in the design specifications by the authors and developers of the technology, but the environment in which tests were actually performed and reported. This classification relies on the scientific literature related to the testing of each gripper to the best of our knowledge. We divided the grippers into three categories:

- Prototypes tested in a laboratory tank: a prototype of the proposed technology was built for validation and testing in laboratory environment (i.e., water tanks), to offer an initial level of confidence for further development.
- Prototypes tested in a pool or in shallow sea water: prototype, system or subsystem modules, in a more realistic or near-final version, were validated or demonstrated in a relevant environment, such as pools or shallow sea water. This includes initial integration at some level with other operational systems, or facing additional design challenges (e.g., control, communication, etc.).
- Prototypes tested on ROVs: a prototype of the technology was as close to the operational version as possible, and was tested in deep-sea, integrating it into an ROV.

## 3. Overview of Marine Technologies for Sampling

An analysis of available technologies for underwater sampling was needed in the perspective of understanding the essential requirements for an innovative underwater gripper for specimen sampling. We are interested in both commonly used solutions for marine sampling, and research status ones. Nonetheless, literature describing sampling procedures is limited [12]. Indeed, in [15], interviewed ROV pilots reported that the choice of the procedure is mostly learned by experience and direct confrontation with the researcher interested in the samples. In fact, video analysis was fundamental to develop a sense of the operational procedures. In Section 3.1, we describe the properties of the tools commonly used in sampling practice: macro-categories of gears for sampling with ROVs and submersibles are presented.

To identify which features are missing in the state of the art of underwater grippers, the research status of new prototypes of underwater grippers was also considered. Most of the prototypes of underwater grippers implement new technologies or properties that demonstrated potential advantages in the underwater environment, such as under-actuation or compliance. Other grippers resulted from water-proofing a well-proven robotic gripper [17]. Just recently, some prototypes claimed the solution of specific scientific sampling tasks [18]. In Section 3.2, we discuss the characteristics of the state of the art of the research on underwater grippers.

### 3.1. Underwater Grippers and Tools Commonly Used for Deep-Sea Sampling

In Clark et al. [12], sampling gears involved in underwater manipulation with ROVs are described. We elaborated and integrated that description with the information gathered from the observation of videos of sampling methodologies [19] and answers to questionnaires proposed to researchers in [15]. According to our analysis, we describe the following categories:

- Grippers, namely the end-effectors of the manipulators, directly used for sample collection;
- Sampling tools, which are manipulated through the gripper and enhance its operating possibilities;
- Storage systems, whose characteristics and conformation influence the sequences of manipulative actions selected.

Within each category, we distinguish between different basic classes of tools according to their properties. In particular, during the interviews in [15], two claws were mentioned (the claw of VICTOR 6000 [20] and the claw of LIROPUS 2000 [21]) that have different properties from the ones mentioned in Clark et al. [12]: consequently, we propose the addition of two classes of manipulator claws: grabber claw and cage claw. Overall, our analysis results in the identification of:

- Four classes of manipulator claws: parallel fingers, opposed or intermeshed fingers, grabber claw and cage claw; details on each class are provided in Section 3.1.1.
- Five classes of sampling gears: suction samplers, scoops and scoop-nets, corers, traps, and water bottles; details on each class are provided in Section 3.1.2.
- Four basic classes of storage systems: bioboxes, carousel jars, ROV racks and baskets; details on each class are provided in Section 3.1.3.

In general, not all ROVs are always equipped with all these systems, and the choice of a specific tool might be driven by its availability on the ROV used in a specific dive, more than its specificity for the collection of the specimen of interest [15]. In fact, the availability of a tool on a specific ROV might depend:

- on the optional tools supported by the vehicle,
- on the resources invested in buying new equipment,
- on the equipment selection during the preparation of the specific cruise.

#### 3.1.1. Grippers: Parallel, Intermeshed, Grabber and Cage Claw

**Figure 1:**
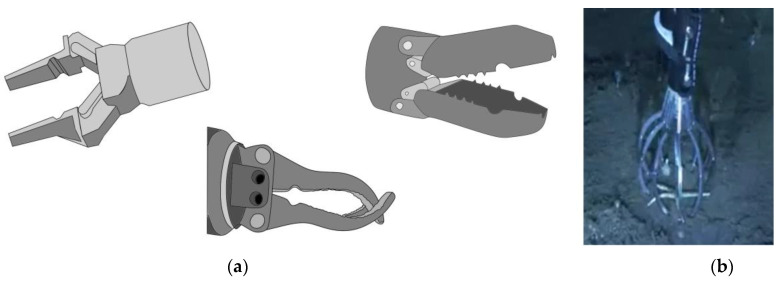
(**a**) From left to right, examples of parallel, intermeshed and grab claw. (**b**) Example of cage gripper: gripper of the ROV LIROPUS 2000. (Reprinted with permission from ref. [22]. © 2022 IEO).

Parallel claws (Figure 1a, left) are grippers whose fingers remain parallel during opening and closing. Parallel claws commonly used in marine sampling are two-fingered, and used for the precision grasping of small samples. Possible damages to the sample may occur when the two internal surfaces of the fingers make contact. To minimize damage, in some cases, the internal surfaces of the gripper are coated with foam, silicon pads, or plastic tubing.

Intermeshed or opposed fingers claws (Figure 1a, middle) are grippers that have round shaped fingers, used to collect whole organisms or delicate specimens. Generally, they have two jaws, with a total of three to four fingers attached to them. The round fingers might be used to directly wrap around spherical or cylindrical tools.

Grab claws (Figure 1a, right) are generally made up of two cupped or dished halves pivoting around a point until their edges join. They allow scooping without the need of a tool, and a grabbing action. The joining edges of the two halves might also perform prehensile grips, but this operation is less straightforward.

Both intermeshed and parallel claws generally have a groove, to firmly hold T-handles of sampling tools (less frequently, grab claws have it too).

Cage claws (Figure 1b) are multi-fingered or grid structures that envelope the sample and often part of the sediment around it, and then part of that sediment is released across the interspaces between fingers or the grid mesh. These claws are not generally thought for power prehensile grasp, but for caging the target, and they are very useful for taking delicate samples in friable sediment substrates.

Claws are made of aluminum, titanium, or stainless steel, which all hold pressure and prevent corrosion. Polyethylene might also be used for claws [8].

#### 3.1.2. Sampling Tools: Suction Samplers, Scoops, Corers, Traps and Niskin Bottles

**Figure 2:**
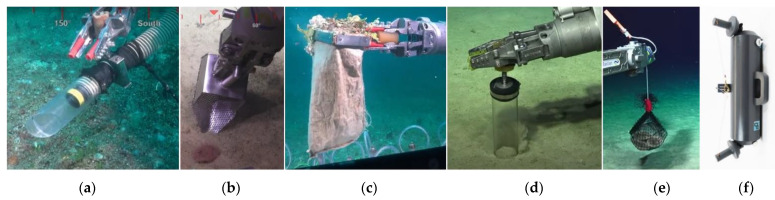
ROV collection tools: (**a**) suction sampler; (**b**) scoop; (**c**) scoop net; (**d**) corer; (**e**) trap; (**f**) Niskin bottle. ((**a**–**e**) Reprinted with permission from ref. [23,24,25,26,27]. © 2022–2021 Schmidt Ocean Institute. (**f**) Reprinted with permission from ref. [28]. © 2022-2022 General Oceanics).

Suction samplers (Figure 2a) are suction pumps ending with a nozzle for sampling the soft body fauna. The tube of the pump is generally flexible, while the nozzle is a hollow ring or tube of a harder material; the nozzle is generally narrower than the tube because it serves as a reference for the allowed dimensions of the samples to be suctioned. This avoids undesired tube clogging, which makes the sampler unusable for the rest of the dive. Suction sampler nozzle optionally has a removable sieve between the nozzle and the tube. When this sieve is inserted, it is possible to suck the sample just to hold it in the nozzle, until the suction power is turned off to release it; when the sieve is not inserted, the sample is sucked down the tube directly to the jar.

Scoops (Figure 2b) are generally used for the collection of the substrate and the small fauna it contains. The content of the scoop is poured into a storage container. *Scoop nets* (Figure 2c) are instead a particular type of scoop made of a bag whose edges are attached to a rigid frame connected to a handle. Pouring is not necessary in case scoop-nets are used, because the bag is also the container in which the sample is stored.

Corers (Figure 2d) are similarly used for sediment collection, and for collecting the fauna which might be contained in the sediment, keeping, at the same time, the sedimentary stratification. It is a tube pressed within the substrate and then gently retrieved. Once loaded, the corer is inserted into a quiver or into the ROV rack.

Traps (Figure 2e) are tools deployed in the sea, accessed by the organisms of interest, and then recovered after a while. Given that traps are released by the manipulator, proper anchor systems are attached to the trap which facilitate the recovery, provided that the dimension of the whole trap fits the containers of the ROV.

Niskin bottles (Figure 2f) or syringe-based systems are used to collect water samples. Bottles have two valves as inputs for the collection, generally kept open until the site of sampling is reached. Bottles can be positioned with the manipulator for collection at specific sampling points, or mounted on a rack on the ROV. When mounted on a rack, bottles can be closed automatically, or by pulling a rope connected to the valves using the manipulator.

Baskets are also used as marine sampling tools. Baskets were reported [15] to be used to scrape brittle specimens with movements that make the sample fall directly into the basket itself.

Finally, collection nets and midwater bucket are two additional tools. Collection nets are used with submersible manipulator mainly after the administration of anesthetizing substances in the proximity of targeted organisms [12]. They are similar to the scoop-nets, but with a larger mesh size, and are generally used for collection in the water columns. Midwater buckets are also tools for the collection of samples in the water column: they consist of a jar with a lid, which can be closed with a rotatory movement as soon as the animals enter them. They are useful for capturing very delicate swimming organisms.

#### 3.1.3. Storage Systems: Bioboxes, Jars, ROV Rack and Baskets

**Figure 3:**
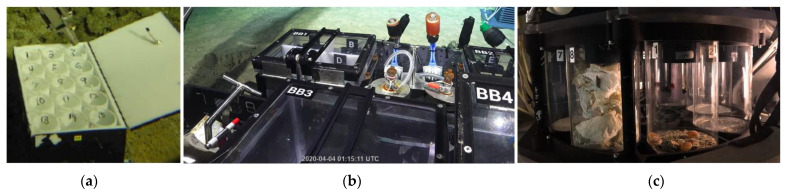
ROV Storage systems: (**a**) biobox; (**b**) ROV rack; (**c**) carousel jar. ((**a**) Reprinted with permission from ref. [12]. © 2022 Wiley. (**b**,**c**) Reprinted with permission from ref. [29,30]. © 2022 Schmidt Ocean Institute).

Bioboxes (Figure 3a) are boxes with covers used to store collected samples during ROV navigation procedures. In general, bioboxes keep the sample in water but without controlling temperature, light or pressure conditions. The boxes should be partitioned and numbered to separate and track samples.

In the case that samples are too large, they are stored directly in the rack of the ROV (Figure 3b), which is an extractable drawer. The rack of the ROV might also host bioboxes, corer quivers, or loaded corers, and scoop net directly: those choices depend on the cruise preparation phase, because the rack of the ROV can be modularly organized according to the foreseen specimen.

The jars receive samples collected through the suction sampler: jars are arranged in a carousel (Figure 3c) that can rotate, allowing a new sample to access a new jar, when needed.

In the case that an ROV is not equipped with a rack, the use of a basket as a storage system was reported [15]. Basket is anchored to the ROV, or held with one of the arms and filled with the other, and recovered once the ROV rises to the surface; the drawback of using a basket (which has no cover, generally) for storage is a higher likeliness of losing the samples.

### 3.2. Research Status of Underwater Gripper Technologies

#### 3.2.1. Studies Selection Procedure

**Figure 4:**
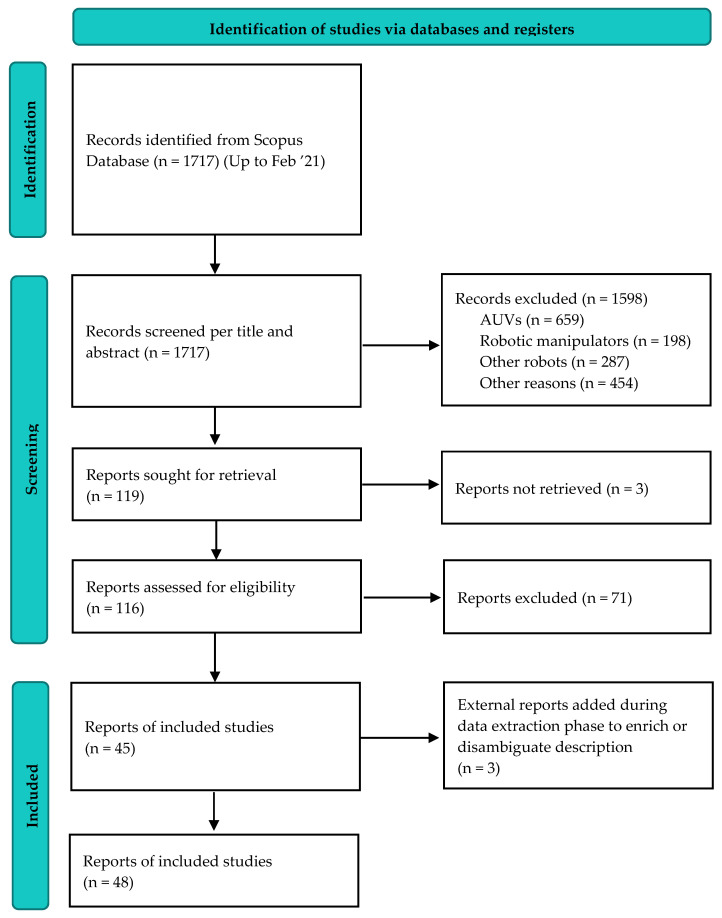
PRISMA flow diagram showing the article screening procedure.

The whole selection procedure is illustrated in Figure 4. The search resulted in a total of 1717 articles. A total of 1645 articles were excluded after title and abstracts screening: 659 were related to autonomous underwater vehicles (AUVs) navigation, control, sensors, localization or object recognition underwater, 198 to robotic manipulators, 287 to other types of robots, 54 to other sea related topics (navigation, diving or fishing), 75 to pure sciences (oceanography, biology, chemistry, etc.), and 325 were excluded for other reasons (e.g., because they were related to education in engineering and marine sciences, etc.).

Overall, 119 articles were selected for full-text reading, but it was not possible to retrieve the full text of three of them. After full-text reading, 71 articles were excluded because they reported primitive concepts of design which were never manufactured or did not account for design issues related to the marine environment (see Section 2.1). Finally, 45 articles were included in the final list, which describe a total of 30 different underwater gripping technologies.

#### 3.2.2. Overview on the Research Status of Underwater Gripper Technologies

**Figure 5:**
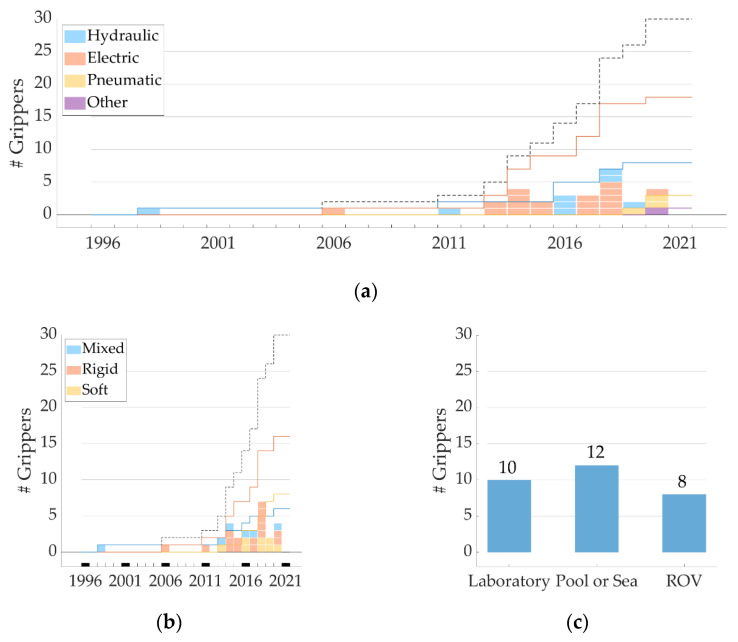
For the 30 grippers identified in this review: (**a**) Number of new grippers per year (1996–2021), grouped by color per type of actuation selected. Solid lines represent the cumulative number of grippers for a given type of actuation over the years. The black dashed line represents the cumulative number of grippers over time. (**b**) Number of new grippers per year, grouped by color per selected technology among mixed, soft, or rigid. Solid lines represent the cumulative number of grippers for a given type of technology over the years. The black dashed line represents the cumulative number of grippers over time. (**c**) Number of grippers tested in each environment: tank in a research laboratory, pool or sea, and mounted on a ROV.

Growing interest has been given to the design of underwater grippers in the last 25 years: Figure 5a reports new underwater gripper technology by year (among the ones identified in this systematic review), considering the year in which the first scientific publication on the gripper was found. The AMADEUS project [31] in 1998 was one of the first actions that fully focused on the development of an underwater gripper. Then, the investigation of different multipurpose configurations registered a growth from 2010 onwards. Figure 5a also shows a prevalence of hydraulic and electrical actuation. Hydraulic actuation is coherent with the actuation type of underwater manipulators: hydraulic actuation is in fact widely employed in commercial systems (e.g., Schilling Robotics, LLC or Hydro-lek, Ltd., among others) [8]. Electrical actuation choice might be again linked to the new trend for the electrical actuation of manipulators (e.g., ECA Group) motivated by the possibility of achieving more precise control [8]. Pneumatic actuation is a recent choice, but during the tests with the prototypes considered here, the pneumatic control system was kept off-board: for a test at a greater depth, either an onboard system would be needed, or a switch to a hydraulic system.

Figure 5b shows the type of technology selected over the years. Most of the gripper prototypes in research status are rigid, but soft technology is emerging as a recent trend.

The bar diagram in Figure 5c divides the grippers according to the testing environment (i.e., tank in a research laboratory, pool or sea, and mounted on an ROV). ROV dives can be very expensive. For this reason, the eventual testing phase on ROV is generally limited to a reduced time slot during campaigns already planned for other purposes. In fact, most of the tests on ROVs reported here involve authors who were affiliated with research institutes owning their own vehicle. Finally, not all the grippers were intended for the use in deep-sea; hence, this could be a second reason why the test in the deep-sea was not considered.

#### 3.2.3. Prototypes Tested in Lab Tanks

**Figure 6:**
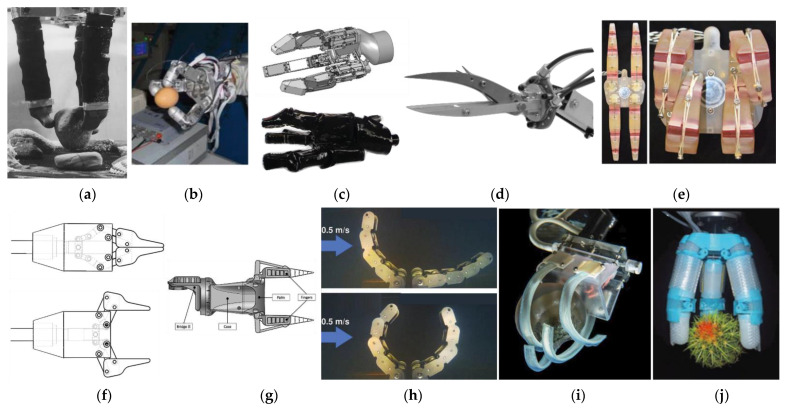
Underwater gripper tested in lab tank: (**a**) AMADEUS; (**b**) HEU II; (**c**) SeeGrip; (**d**) Okinawa; (**e**) Stanford; (**f**) Malaysia Pahang; (**g**) Calabria; (**h**) Tokai; (**i**) Wyss Ultragentle; (**j**) VSPP-3. ((**a**) Reprinted with permission from ref. [31]. © 2022 Emerald Publishing Limited, all rights reserved. (**b**,**e**,**f**,**h**) Reprinted with permission from ref. [37,42,46,49]. © 2022-2020 IEEE. (**c**) Reprinted with permission from ref. [38,51]. © 2022 IEEE and © 2022 DFKI. (**d**) Reprinted from ref. [41], CC-BY license. © 2022 Fuji technology press. (**g**) Reprinted with permission from ref. [47]. © 2022 Elsevier. (**i**) Reprinted with permission from ref. [18]. © 2022 AAAS. (**j**) Reprinted with permission from ref. [50]. © 2022 Liebert Pub.).

Table 3 reports the main characteristics of the underwater research grippers that were tested in laboratory, while Figure 6 shows a picture or a representation of the systems. In the following, we briefly discuss each gripper technology.

AMADEUS (Figure 6a) [31,32,33,34,35] is a gripper intended for dexterous manipulation which incorporates force and slip sensors. It exploits deformable metal bellows to meet the need of compliance, but is sustained by a rigid skeleton to achieve a kinematically determined structure. The movement is a result of the differential of pressure in three parallel actuators that constitute a finger. The AMADEUS project involved a strong effort in developing sensors for the fingertip, using strain gauges and slip sensors encapsulated in silicone rubber, and in the implementation of a hierarchically structured control architecture. AMADEUS has started, since 1998, two of the trends that would have taken root in the subsequent decades: on one hand, the tentative of employing compliant technology, hampered by the complexity in achieving control. On the other hand, the importance of adding waterproof and pressure insensitive tactile sensors to achieve better control in manipulation.

The HEU II hand (Figure 6b) [36,37] replaced the cables that drove the HEU I hand with geared actuation. Three rigid fingers are anchored to a frame and spaced at 120°, and fingertips have sensors to measure the 6-axis force torque. Only finger positioning was tested underwater without assessing the grasping capabilities, although the grasping of various objects was evaluated with different configurations in air. The possibility of ab/adducting the fingers enables both circular and parallel configuration of the fingers, but testing underwater was not that extensive.

SeeGrip project (Figure 6c) [38,39,40] developed a gripper with fast opening/closure and repeatable precision. The gripper was actuated by passively compliant hydraulic piston that exploited 4 valves per finger. Insertion of sensors on the fingers was fostered by combining technologies as strain gauges, piezoelectric and fiber optic sensors. SeeGrip project focused also on adaptive control of the gripper. The whole system was covered with an oil-filled skin glove, and tested in a 600 bar tank gripping a spherical handle to simulate operations at 6000 m depth. Even if no tests in the sea were performed, the strength of the SeeGrip resides in having comprehensively considered multiple aspects in the design, as actuation, control, sensing, grasp configuration, waterproofing and tests under pressure.

Okinawa University developed an underwater manipulator [41] for collecting crown-of-thorn starfishes, whose outbreaking massively destroyed coral reefs. The manipulator has a scissor-like end-effector (Figure 6d): one of the two fingers is fixed, and the other arched. Fingers have sharpened edges, inspired to the tongs used to collect crown-of-thorns starfishes, so that they can enter narrow spaces. Payload of the system and pressure resistance was tested. The technology is easy, and tests with the organisms have not been performed yet, but the approach adopted strongly considers the task to be performed in the definition of the specifications.

Stanford University developed a hand (Figure 6e) [42,43,44,45] that exploits flexural hinges as joints, conferring compliance to the fingers during the grasp. The peculiarity of this hand is the addition of suction flow on the tip, to counteract the typical force that pushes the object away during an underwater grasp while fingers close. The hand closure is tendon driven and the opening exploits return springs. The hand allows for pinching, but also for intermeshed fingers envelope grasp, because one among the pair of opposed fingers has a slightly twisted position. Thus, when large forces are applied, this configuration allows the two opposed fingers to slide past each other.

Bioinspiration from the crab was the guide for the development of the Malaysia Pahang University gripper (Figure 6f) [46]. Authors made a robust gripper that works as foot leg for point walking when it is closed, and gives the possibility of gripping an object when it is not used as a foot.

University of Calabria developed an underwater manipulator [47] implementing also a gripper (Figure 6g) with interchangeable palm that enables switching between 2- or 4-fingered configuration. It has an actuator for wrist rotation and one for closing the gripper, and the closing mechanism consists of the first phalange stopping as soon as an object is encountered, while the second phalange wraps around it.

A rigid gripper for envelope grasp was developed by Tokai University (Figure 6h) [48,49]. The movement of the two opposed fingers starts from a timing belt guiding the gears, which drives a series of 4 joints that envelope the object, adapting to it. The tip-rollers of each of the two fingers receive the rotational motion too, so they can pull up a pinched object, transferring it to a stable envelope grasping. A tension spring mechanism guided by a motor adjusts the rotation stiffness of the proximal gears. The possibility of mechanically adjusting the stiffness of each gear limits the need of sensors in the fingers, and consequently eases the procedure of waterproofing the design.

Wyss Institute developed a prototype of gripper (Figure 6i) [18] aiming at the reduction of the stress on animals. With a soft “ultra-gentle” actuator, live specimens of jellyfishes were collected in a lab tank, successively demonstrating that the gentler grasp of this gripper stimulated a reduced physiological stress response than conventional ROV grippers [52].

VSPP-3 gripper (Figure 6j) [50] is a prototype made of pneumatic joints linking rigid supports, which hold jamming components. Those components offer the interesting feature of compliance to spines, that worked even when the jamming membrane was pierced because the jamming component does not need tightness. On the other hand, improvements on the design are needed to test it at depth, because some components were glued during assemblage, and likely, gluing will not withstand prolonged salt water exposition, acid or hot temperature environments (e.g., depending on geological site activity), and overall pressure.

#### 3.2.4. Prototypes Tested in Pools or the Sea

**Figure 7:**
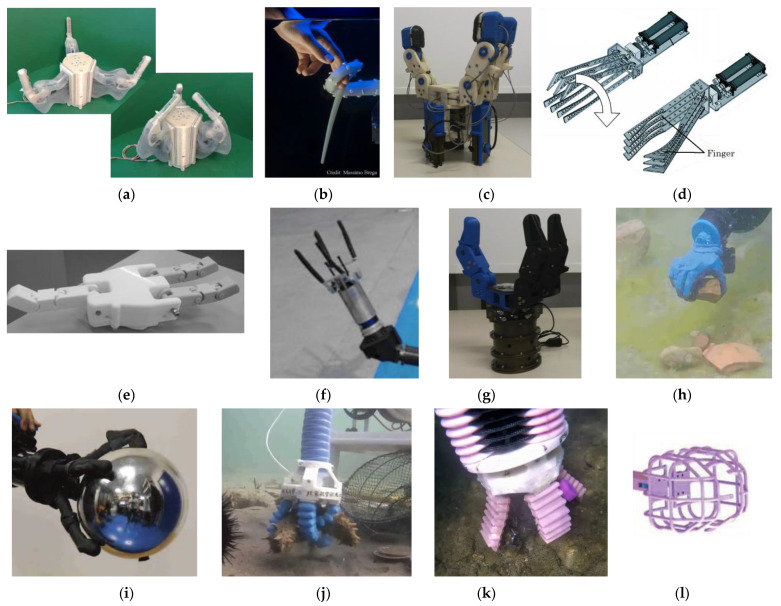
Underwater gripper tested in pool or sea: (**a**) TRIDENT-Skin; (**b**) PoseiDRONE; (**c**) TRIDENT-UNIBO; (**d**) ARTEMI; (**e**) GUH14; (**f**) UJIOne; (**g**) MARIS; (**h**) IIT SoftHand; (**i**) IIT Gripper; (**j**) OBSS; (**k**) Silver; (**l**) Tshingua. ((**a**,**e**) Reproduced with permission from ref. [53,61]. © 2022-2015 International Federation of Automatic Control (IFAC). (**b**–**d**,**f**–**i**,**l**) Reprinted with permission from ref. [17,54,57,58,60,66]. © 2022-2020 IEEE. (**j**) Reprinted with permission from ref. [63]. © 2022 SAGE. (**k**) © 2022 Scuola Superiore Sant’Anna Pisa).

Table 4 reports the main characteristics of underwater grippers that were tested in pools or the sea, while Figure 7 shows a picture or a representation of the systems. In the following, we briefly discuss the technologies.

TRIDENT project created three different grippers. The first, here referred to as TRIDENT-Skin (Figure 7a) [53], has a rigid structure, and a deformable skin to be filled with oil for operation at depth. The deformability of the skin offers the possibility of balancing internal oil pressure and external water pressure, and the design of the skin is thought to minimize gripper internal volume variation between opening and closing configurations. These features limit the possibility of undesired water intrusion. The second gripper, UJIOne (Figure 7f) [54], could perform envelop grasps and hooking manipulations, and the self-amalgamating tape that fixes the sensors also offers friction that helps to achieve a stable grasp. It was conceived as simple solution for substituting the third gripper, UNIBO hand [54,55], during the development phase. UNIBO hand (Figure 7c) has an opposed thumb and two fingers capable of ab/adduction, so that it could achieve circular and parallel fingers configurations. Under-actuation simplifies the control: joints are coupled per type (adduction, proximal flexion, and distal flexion joints) and are driven by a single actuator per type, by means of a closed loop cable transmission.

The MARIS project brought some amelioration to the results achieved with the TRIDENT project: MARIS gripper (Figure 7g) [56,57] preserves the kinematic structure of the UNIBO hand, but the dimensions and weight were reduced, and motors moved to an independent slot for fast substitution. Sensors (wrist F/T, camera, lights) were integrated in the gripper.

The PoseiDRONE gripper (Figure 7b) [58,59] was inspired by octopus tentacles, that have both locomotion and manipulation abilities. The gripper is a silicon cone actuated with tendons, whose arrangement regulates bending directions. It can envelop and hold objects. This gripper has the advantage of adaptability to objects shapes, and it is useful to anchor to a pipe for example, but the usage of tools might become difficult due to the complex kinematics of soft structures.

ARTEMI [60] was conceived as a gripper robot. In fact, excluding the locomotion system, it consists of a simple gripper (Figure 7d) with only two degrees of freedom (DoFs): wrist rolling and gripper opening/closure. The fingers profile is curved to better fit grasped objects, and rubber was added to the fingers to prevent slippage.

GUH14 (Figure 7e) [61] is a gripper with three independent intermeshed fingers, where gears drive the proximal phalange and tendon transmission drives the distal ones. The driving components are integrated in the palm of the hand. This reduces the total volume of the gripper, allowing use also on small ROVs.

IIT presented two types of grippers [17]; one (Figure 7i) targeted for grasping large objects, and another, the IIT SoftHand (Figure 7h) for finer manipulation, because of the possibility of performing power, pinch and lateral grasp. Both grippers are compliant and underactuated. Noteworthy is the tool-changer system based on magnetic coupling, which enables rapid switching between the two grippers.

Tshingua University developed an underwater vehicle manipulator system (UVMS) with a lightweight gripper (Figure 7l) [66]. The gripper was made of two halves of a cage, which can close by pivoting around a common axis, to collect marine species. The maximum force that the gripper is able to exert was limited by thresholding the current absorbed by the motor: this is needed to avoid crashing the sample. They proved the successful collection of sea urchins, cucumbers and scallops on the seabed. Nonetheless, because of the shape of the cages, a more irregular surface of the substrate might represent an obstacle for collection. Moreover, collection with a caging mechanism could be less straightforward when samples require being detached.

The last two grippers tested in a sea environment are both soft and pneumatically actuated. Opposite-bending-and-stretching structure (OBSS) (Figure 7j) [62,63,64] was a manipulator-gripper system which demonstrated the successful collection of marine organisms as sea cucumbers or sea urchins. A similar soft manipulator-gripper system (Figure 7k) can be found integrated into the Silver crawler robot [65]. Soft technology is intrinsically compliant and adaptive, but both the systems still need to integrate the pneumatic controllers, that were by now kept off-board during sea trials.

#### 3.2.5. Prototypes Tested in the Deep-Sea on ROVs

**Figure 8:**
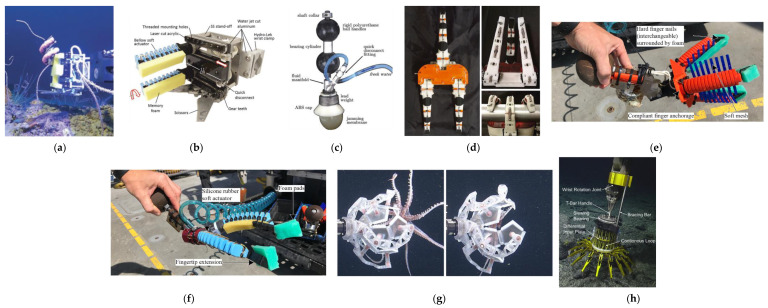
Underwater gripper tested on ROVs: (**a**) Wyss Boa, (**b**) Wyss Bellow, (**c**) Universal Jamming gripper, (**d**) Ocean One, (**e**) Wyss 3DP, (**f**) Wyss Bellow v2, (**g**) Wyss RAD (**h**) JPL-Nautilus. ((**a**,**b**) Reprinted from ref. [10], CC-BY license. © 2022 Liebert Pub. (**c**) Reprinted with permission from ref. [72]. © 2022 Liebert Pub. (**d**) Reprinted with permission from ref. [76]. © 2022 SAGE. (**e**,**f**) Reprinted from ref. [68], CC-BY license. © 2022 PlosONE. (**g**) Reprinted with permission from ref. [69]. © 2022 AAAS. (**h**) Reprinted with permission from ref. [77]. © 2022 Wiley).

Table 5 reports the main characteristics of underwater grippers tested in the sea on ROVs, while Figure 8 shows a picture or a schematic representation of each system. In the following, we briefly discuss the technologies.

The Wyss Institute presented four designs of soft grippers and a fifth design of gripper configured as a cage; those prototypes were all tested on the ROV. Firstly, they presented hydraulically driven actuators called “boa-type” and “fiber reinforced bellow-type”, tested to handle pressure up to a depth of 800 m. Actuators were coated with a layer of foam to enhance their compliance, and integrated on a palm that allows for different mounting configurations. This palm also mounted a scissor system, directly actuated with the push–pull rod of the arm system.

The boa-type actuator gripper (Figure 8a) was used to perform envelope grasp, and tested for the collection of a whip coral [10,67]: a peculiar result is the coiling effect of the actuator that reduces the impact of eventual suboptimal positioning. The bellow-type actuator was instead used as finger of a four intermeshed fingers gripper (Figure 8b) [10,67], and gently closed around a soft coral specimen without damaging it.

The same bellow-type actuators were used in other gripper versions, in which fingers assume circular configurations with 2, 3, 4 or 5 fingers [67,68]. The two-fingered version (Figure 8f) was also endowed with a rigid nail covered with soft foam, which enables it to perform a pinch grasp. The bellow-type gripper also inspired a new design that can be 3D-printed on-deck (Figure 8e) [68]: the concept of using rapid prototyping during the cruises highlights the need for the adaptation of the design to the needs identified during the dives. These 3D-printed actuators were enriched with a soft mesh to increase contact point and avoid the sample escaping between fingers.

Finally, the Wyss Institute Rotary Actuated Dodecahedron (RAD) gripper (Figure 8g) [69] is a flat structure that is folded by the external linkages system to form a dodecahedron, which can enclose free-swimming/floating specimens. The folding is driven by a rotary actuator tested for 11 km depth. The gripper was demonstrated during the collection of squids and jellyfishes up to 700 m in depth [69]. Wyss Institute recently developed the RAD2 [70], a second version of the RAD integrating a tissue sampling system that preserves samples in situ (i.e., for genomic sequencing).

Jamming technology was also brought underwater, using water as fluid media and glass particles: Licht et al. [71,72,73] developed a Universal Jamming gripper (Figure 8c) characterized by a membrane partially filled with particles and sealed to a rigid conical cap. The membrane was kept free from the rigid cap to avoid the cap exerting a pushing force on the object through the membrane. Three grasping configurations were identified according to the shape of the object: interlocked, suction, and friction. This gripper was tested at 1200 m depth, with various objects positioned on the ROV rack.

The Ocean One hand (Figure 8d) [74,75,76] is an evolution of the Stanford hand [42,43,44,45], conceived for the Ocean One avatar, which aims at substituting human divers in underwater manipulation. As for the Stanford hand, the Ocean One hand consists of rigid parts, covered with pads that increase friction, flexural hinges as joints, and preloaded return springs. In addition, tendons were doubled for additional strength, and over-designed to avoid failures. A mechanism to select between two degrees of transmission stiffness was also implemented, to obtain different grasping behaviors by operating the motor in opposite directions. The Ocean One hand was tested on the avatar up to 91 m depth. In Nadeau et al. [45], the design of the Ocean One finger is used to test fingertip suction flow as an aid for objects handling.

The JPL-Nautilus gripper (Figure 8h) [77] was conceived to allow anchoring to curved surfaces with micro-irregularities, but it could also collect rock specimens up to 2000 m depth. It is actuated by impressing a rotation to its T-bar handle (requiring no supplementary supply), and the opening and closure are regulated by tension and extension cables. The gripper is efficient for anchoring purposes, and enrichment with the corer system in the center of the system is foreseen. However, since the gripper is bulky, the workspace and the manipulability of the arm are reduced.

## 4. Discussion

In Section 4.1, we summarize the trends in the research status on design of gripper technologies for underwater sampling. In Section 4.2, we analyze the possibility of performing each specific manipulative action with the technologies that we presented. In Section 4.3, we point out the environmental and operational requirements the authors considered while setting the specifications for the design of underwater gripping technologies.

### 4.1. Trends in Gripper Technologies for Marine Sampling

The differences between the grippers and tools that are commonly used in marine sampling and the ones in the research status are substantial. Commonly used grippers, in particular the parallel, intermeshed, and grabber claws, resemble the shape of existing general industrial grippers, whose potential was extended by the possibility of easily interfacing them with tools that are very similar to the ones used by humans, such as scoops, baskets, collection nets, and so on. The research statuses of prototypes of underwater grippers focus on solving specific issues related to underwater manipulation, but they are more likely to fail in scenarios that differ from the ones that they were designed for.

Since the research on the design of underwater gripper started, the under-actuations of multiple joints and tactile sensing restoration underwater were topics of greater interest. The under-actuation and selection of proper transmission systems (among which tendons are pretty common) are fundamental to:

- avoid a design with a high number of dynamic seals, since their wear rate increases for high-pressure applications;
- keep the weight as proximal as possible to reduce inertial effects.

Tactile sensing is instead a core aspect for control systems that includes feedback, but again inserting electronics in the very distal parts of the hand requires careful waterproofing, pressure independent sensors calibration, and organization of the signal channels. More recently, mechanical methods for selecting the stiffness were used to avoid the need for tactile sensing [48,76].

Bioinspired prototypes emerged to solve specific issues, keeping an organism as a source of inspiration for the solution [46,58]. Other technologies were inspired by tools already used (as tongs) for the manipulation of a specific organism, e.g., starfishes [41], jellyfishes [18], and so on.

Recently, the research trend is increasingly oriented to soft solutions: this technology allows one to exploit mechanical compliance to comply with misplacement, but failures appear more frequently. Soft materials as silicone rubbers are frequently employed because of their resistance to the variation of environmental factors (such as temperature and acidity), but they are generally characterized by lower strength (such as tensile and fatigue strength) [78], which might result in tearing after a number of cyclic pressurizations [18]. Efforts are directed toward the optimization of the design of soft prototypes: on one hand, mechanical properties are improved by reinforcing rubbers (e.g., by embedding fibers) and simultaneously avoiding tearing between extensible components and less extensible ones [78]; on the other hand, the maintenance process is kept simple to allow component substitution. Moreover, to preserve a certain degree of compliance in the soft solutions, the internal vs. applied pressure relation has to be well characterized.

Jamming is also explored as variable stiffness technology for gripping [72], or demonstrating resistance to perforation [50].

Soft technologies are mostly hydraulically actuated. Few solutions are pneumatically actuated [50,63,65], but before being able to bring them to a depth, there is the need to embed an underwater pneumatic controller on the ROV.

It is worth mentioning that a certain degree of compliance was considered since the conception of the first innovative gripper, within the AMADEUS project [31]. Similarly, traditional grippers benefit from compliant pads made of silicone; rubbers of high-density foams. The limited amount of sensory feedback available in the underwater environment motivates the shift from pure closed-loop control of the grasp, to closed-loop plus mechanical interface. This might partially explain the growing interest in soft technologies for underwater applications.

A more recent trend pays attention to solving specific aspects of the sampling, and evaluates the technology relatively to the specific aim (rather than validating the technology for use underwater, as in the past). Thus, metrics considered for assessing the technology evaluate how much the condition of the organism before sampling is preserved [18,52], up to achieving systems that directly perform sample preservation in situ [74,76].

### 4.2. Versatility of Gripper Tools in Covering Atomic Manipulations

In [15], we analyzed reports of sampling procedures performed with ROVs, provided by researchers or ROV pilots, to extract the series of actions performed during sample collection procedures. We defined *atomic manipulation* or *atomic action* as the single specific interaction of the manipulator with an object (e.g., break a coral fragment, insert the coral fragment into the biobox, etc.). Then, from the analysis of the series of atomic manipulations, we built a taxonomy of atomic manipulations. Atomic manipulations identified in [15] are reported in black in Table 6.

In Table 6, we compare each gripper technology (both commonly used for underwater sampling and research prototypes) with each atomic manipulation, in order to evaluate whether the gripper or tool offers a specific action possibility.

While performing this comparison, we observed that research status grippers were tested for additional atomic manipulations, that were not originally present in the taxonomy that we built in [15]. These additional atomic manipulations are: *Suction on*, *Suction off*, *Scissor cut*, *Lever*, *Hook*. We report those atomic manipulations in red in Table 6. *Scissor cut*, *Lever* and *Hook*, were labeled with progressive numbers *13.*, *14.* and *15. Suction on* and *off* performed with the sole gripper (that is without the use of a tool) are just slight modified versions of the actions *Suction on* and *off with tool* (atomic manipulations *7.A* and *9.A*) that we had already retrieved from the analysis of common sampling procedures in [15]. For this reason, *Suction on* and *off* performed without additional tools were numbered as atomic manipulations *7.* and *9.* In fact, “A” indicates that the gripper holds a tool to perform the action. In [15], more information is given on the notation used to number the atomic manipulations.

We assigned the possibility of executing an action to a gripper if it was tested, or if there was clear evidence of the applicability of an action in terms of gripper shape, morphology and compliance, which could be inferred from the available descriptions. White spaces represent ambiguities that might be solved by testing the actions. Patterned columns represent analogous ambiguities, whose successful resolution is subordinated to the capacity of the gripper to firmly hold a tool. Generally, tools used in underwater sampling procedures present handles, and most of the grippers have the proper fixture to hold it. The ambiguities in patterned spaces could be solved if the ability of a gripper to rigidly hold a particular handle shape (i.e., the T-bar handle) is demonstrated.

Commonly used gripper categories either focus on gripping action or caging action. The possibility of stably holding a tool enables supplementary action such as scooping, coring, or suctioning. Intrinsic resistance to the impact of the gripper affects the efficacy in performing pushing actions.

Soft or intrinsically compliant grippers were not judged suitable to perform pushing or lever actions. Rigid grippers with at least a finger or flat component should enable scraping, while grippers whose entire structure consists of stiff materials were considered suitable for pushing or *Lever*. Almost all the prototypes were tested for grip and release. The *Grip and pull* action is potentially possible whenever the technology is able to grip and the supporting vehicle is able to exert pulling force. Nonetheless, we reported the gripper to be able to perform this action only when at least a pulling test was reported. The *Grip and twist* action might be possible whenever the technology is able to grip and the supporting vehicle or the gripper allows a wrist-like rotation. However, we reported the gripper to be able to perform this action only when at least a twisting test was reported. As concerns tool use, we reported that the gripper is able to hold a tool only when at least a test concerning manipulation with tools was reported.

Some soft grippers were tested only for caging (as the Ultragentle gripper [18]), others both for caging and gripping (as the gripper of the Silver [65], or the Wyss Bellow type v2 [68], which can cage an organism or pinch it by means of the rigid nails). Rigid grippers thought to be for caging (as the Tshingua gripper [66]) are generally not thought of for pinch grip, because of the risk of squashing the animal.

Finally, the Stanford hand [42] implemented itself a suction mechanism, so it is able to perform suction actions without the need for a tool: the strategy of integrating tool capability directly in the gripping system might reduce the time needed for changing the tool and improves manipulation performances.

### 4.3. Environmental and Operational Requirements and Possible Solutions

In a harsh environment such as the marine one, foreseeing all reasonably difficult aspects is a key in the design. A commonly accepted design standard for specific environments suggests the guidelines to refer to. Moreover, knowledge of the properties and limitations of the ROVs impacts the design choice. This section discusses which environmental and operational requirements were specifically and recurrently considered in the development of gripping technologies for use in the deep-sea. Environmental requirements are related to proper choices that guarantee functioning underwater. Operational requirements tackle the compatibility of the system with the supporting structure, that in this case, is the ROV. These requirements can be integrated to the task-related ones when referring to a specific application.

Some of the mentioned gripping technologies were prototypes, and therefore manufactured with common rapid prototyping materials. On the long-term use, perspective authors highlighted the importance of using materials that are resistant to sea water salinity and to corrosion. The selection of the materials should also consider the need for overall robustness against impacts. This need comes from the fact that the end-effector is the most frequent site of contact (intentional and accidental) with the environment. Intrinsic mechanical compliance in the system might help in this respect: this could be achieved with back-drivable transmission systems or by decoupling the system from the motor (i.e., magnetic coupling [17]). Compliance control would be another possible solution, if a reliable force sensing system was available.

Sensing, especially a tactile one, is also a key element for automatic grasping force control. However, in this case also, it is difficult to achieve reliable tactile sensing underwater. First, because there are few available tactile sensing solutions for underwater environment (which were reviewed in [14]), and only part of them was tested under high pressure. Moreover, tactile information obtained from underwater tactile exploration was reported to be of lower quality than the one obtained from exploration in air [79], hence they might require appropriate processing before being able to use them for grasp force control.

Control is also generally employed to limit environmental disturbances such as currents and buoyancy, but in order to keep the control as simple as possible, the recommendation is to orientate the design toward neutral buoyancy, and lightweight to limit the inertial effects on the manipulator. Moreover, buoyancy effects depend on volume variation. Consequently, when deformable bellies are employed, limiting the variation of volume between different configurations of the gripper (i.e., opened and closed configurations) is important in order to achieve more precise modeling of buoyancy effects (as underlined in [53]).

In the deep water, a designer should obviously consider sealing and pressure compensation. A trending approach is to prioritize the isolation of water or pressure sensitive components, leaving the more resistant ones in contact with water. Sealing solutions should account for maintenance and the possibility of component substitution.

As suggested by Stuart et al. [43,44], the finger closure dynamics in a fluid introduce a pushing action toward the object to be gripped. A specific requirement for gripping applications suggests counteracting this action.

Finally, given the supporting vessel specifications, a gripper should comply with those specifications in terms of: physical connecting interface; actuation type; physical interface and communication protocols of control and sensing architectures; and finally, the efficient exploitation of supply resources.

In [15], we provide a more formal list of environmental and operational requirements for the design of an underwater gripper. Considering the list of actions in Table 6, we also add the task-related requirements for the design of an underwater gripper that is intended to perform the task of sampling marine organisms (or part of them) with an ROV.

## 5. Conclusions

Underwater telemanipulation for sample collection is performed nowadays using manipulators designed to perform marine heavy industrial tasks. Moreover, achieving the needed level of dexterity by controlling those manipulators is complex and increases the operator’s workload. The solution to a design problem starts from the clear understanding of the required functionalities and the conditions that it should work in, but sample collection procedures are scarcely described in the literature. Those functionalities surely found a partial solution in the state of the art of the research, that has to be considered as well.

Within this review, we described the grippers and tools commonly used in underwater sampling practice. We compared the peculiar solution encountered by reviewing the state of the art of underwater grippers for dexterous manipulation, with the solutions in use. We analyzed the common design solutions, and pointed out whether a technology covers the typical manipulative actions performed during sampling procedures or not. In this analysis, we considered both the research status prototypes of underwater grippers and the gripper categories that are commonly used for marine sampling (which were often originally conceived as end-effectors of underwater industrial manipulators), but we did not comprehensively explore the industrial solutions. Future developments of this work might review industrial solutions by searching for patents and screening commercially available grippers. Finally, we discuss the main environmental and operational requirements for an underwater gripper, that will be merged in the second part [15] of this series of papers with the task-related requirements.

Solutions have been designed to satisfy specific actions, but to extend the possibilities of a single technology, additional tools are adopted, but tool changing according to the needs requires time. A significant limit to the technological advancement of this kind of systems could also be the difficulty in accessing the depth of the sea for testing the devices. Accessibility is in fact almost limited to owners of ROV or analogous vehicles, and expensive: experiencing the possible situations one can encounter is fundamental, together with the ability of foreseeing them. This review is our attempt to integrate the limited amount of time that can be stolen for testing during ROV campaigns, with an earlier thorough discussion on the issues of design for underwater, and an analysis of accurate and varied evidence that research can provide.

## Figures and Tables

**Table 1:** Search query strings. ALL means that search is made through the whole text of the article, ABS stands for abstracts, KEY stays for keywords, SRC TITLE stands for title of the source (journal, conference or book), double quotes are used to search for the exact string; the star symbol * represents any character, allowing the search for any word with a specified prefix. MTS stands for Marine Technology Society.

	String 1	String 2
ALL	gripper OR manipulation OR finger OR hand	
	AND	
TITLE-ABS-KEY	underwater OR deep-sea OR marine OR subsea	
	AND	
TITLE-ABS-KEY	robot* OR “end effector”	-
SRC TITLE	-	robot * OR (ocean * AND engineering) OR (marine AND technology) OR (autonomous AND vehicle) OR (mts AND ocean)

**Table 2:** Inclusion and exclusion criteria considered in the selection of articles based on abstract and title reading.

Inclusion Criteria	Exclusion Criteria
Design of grippers explicitly purposed for use underwater, or tested for use underwater	Design and modelling of grippers whose potential application underwater is proposed but not considered in design or test phase
Control architectures and algorithms for underwater grippers	Decisional algorithms for task planning for underwater autonomous manipulation.
Sensing for underwater grippers	Design, modelling, control and sensing for manipulators, AUV or other type of robots
Object sampling underwater for scientific purposes	Pure sciences articles (Engineering, Biology, Chemistry, Sea Sciences, etc.)
Underwater manipulation for scientific purposes	

**Table 3:** Underwater gripper tested in laboratory tanks. Legend: # (Number of), n.a. (not applicable). Weight: F (Finger weight). Dimensions: L (Length), W (Width), D (Depth), LF (Finger length), WF (Finger width). Force: P (Peak), NO (Normally operating). Actuation: H (Hydraulic), E (Electric), P (Pneumatic). Sensors: F (Force), P (Position), H (Pressure), C (Contact). Sealing: D (Drive), T (Tensioning), M (Mechanics), S (Sensors). Tested Depth: LT (Lab tank), S (Still), P (Positioning), G (Grasping). Tested objects: O (Out of water), W (in Water).

**Gripper**	**Ref.**	**Year**	**Technology**	**#Fingers**	**#Joints**	**#Actuators**	**Weight**	**Dimensions**	**Finger Movement**	**Force/Torque/Speed**
AMADEUS	[31,32,33,34,35]	1998	Mixed	3	6	1	3.5 kg	L 365 mm	20 mm	P: 15.45 N
HEU II	[36,37]	2006	Rigid	3	9	3	-	FL 130 mm	Joints: 75°, 40°, 75°	0.35 Nm, 1 Nm, 1 Nm
SeeGrip	[38,39,40]	2011	Rigid	3	6	1	-	FL 297 mm	-	-
Okinawa	[41]	2014	Rigid	2	2	2	-	L 200 mm	Roll: 360°, Open: 90°	5.9 Nm/28.0 rpm
Stanford	[42,43,44,45]	2014	Mixed	4	12	4	-	-	-	-
Malaysia Pahang	[46]	2017	Rigid	2	2	1	-	-	-	40 ÷ 180 N
Calabria	[47]	2018	Rigid	2 or 4	5 or 9	2	-	LF 130 mm	Joint 3: 90°	-
Tokai	[48,49]	2018	Rigid	2	8	4	F 1.5 kg	LF 216 mm, WF 83 mm	-	-
Wyss Ultragentle	[18]	2019	Soft	6 (or 4)	n.a.	1	123 g	L ~150 mm Palm W 78 mm D 45 mm	-	NO: 0.0455 ± 0.007 kPa by each actuator
VSPP-3	[50]	2020	Mixed	3	2	2	460 g	L 130 mm, Ø 170 mm	-	<5 N per finger
**Gripper**	**Actuation**			**Transmission**				**Sensors**		
AMADEUS	H: Fixed displacement gear pump			Pressure reducing valve, control valves (9 to the bellows, 1 to knuckle j.), metal bellows, knuckle joints, cardan joints				F: Strain gauges (tip); P: Potentiometer (knuckle j.); H: Pressure transducer (act.); C: PVDF (slip).		
HEU II	E: DC Servomotor, Maxon			Bevel gear				F: Strain gauges (tip); P: Hall effect rotary encoders (j.)		
SeeGrip	H			Control valves, piston, parallel linkage				F: Strain gauges; P: Absolute encoders; C: Piezoelectric (texture, slip) and optoelectronic (distribution).		
Okinawa	E: DC geared motor			Tendons, worm gears, magnet coupling				P: Rotary encoder.		
Stanford	E, Return springs			Pulley, tendon				F: Hall sensors and magnet; C: Estimate from suction flow		
Malaysia Pahang	E: DC Indirect drive motor			Puller shaft and scissoring mechanism				F: Estimated from load current		
Calabria	E: Servomotor			Gear wheel and crown wheel (wrist). Self-locking mechanism (finger): worm screw and two couples of gear wheels				-		
Tokai	E: DC micromotor, 24 W, Faulhaber 2342S024CR			Drive: Timing belt, diff. gears mechanism Tensioning: wire spring				C: Estimated from the angle of the base gear		
Wyss Ultragentle	H			Control valves, Tubing				-		
VSPP-3	P			Tubing				-		
**Gripper**	**Sealing Measures**			**Target**				**Test Depth**	**Tested Objects**	
AMADEUS	D: Oil filled case, O-ring S: Silicon embedding, O-ring (connector)			Obj. 10 ÷ 150 mm				LT, G	O: Metallic cylinder, can W: Rock, 75 mm in diameter and less than 1 kg	
HEU II	O-ring (silent seal); Silent ring, bevel and bush (shaft, bearing)			-				LT, P	O: Tennis ball, egg, pen, card, bottle, screwdriver	
SeeGrip	M, S: Oil filled glove			Spheres or cylinders, apple-sized				LT, G	O in 600 bar tank: Sphere Ø 8 cm	
Okinawa	D: Pressure tight case			Starfish collection				LT, G	W: Payload of 3 kg	
Stanford	-			Precision pinch (small obj.) and secure envelop grasp (tools)				LT, G	W: Lego Block, PVC cylinder Ø 5 cm	
Malaysia Pahang	M: Aluminum parts			Obj. 50 ÷ 200 mm				LT, G	W: Metallic cylinder, prism and plate, 30 ÷ 60 g	
Calabria	D: Case enclosure and O-ring			-				P	O: Ball, plastic prism W: Cylinder, Carafe <1.5 kg in water	
Tokai	D: Magnetic coupling T: Gasket, o-ring, watertight bulkhead			Envelope grasp, stability against current. Obj. Ø 120 mm, 100 g				LT, G	W: Bowl Ø 13 cm	
Wyss Ultragentle	-			*Aurelia aurita*, *Catostylus mosaicus*, and *Mastigias papua* (7 to 10 cm) jellyfishes				LT, G	W: Silicone synthetic jellyfishes, *A. aurita*, *C. mosaicus* and *M. papua*	
VSPP-3	Glue			Compliance to spines, even if pierced				LT, G	W: Durian, cactus, pineapple, pitaya, apple, grape, egg, cherry, cabbage, eggplant, drill, plier, hammer, Rubik cube and a pyramid	

**Table 4:** Underwater gripper tested in pools or sea. Legend: # (Number of), n.a. (not applicable). Dimensions: L (Length), W (Width), D (Depth), LF (Finger length). Finger movement: x and y represent the range of movement of the fingertip along x and y orthogonal axis, x is parallel to the palm, y is perpendicular to the palm. Force: L (Lift), P (Pinch), Po (Power). Actuation: H (Hydraulic), E (Electric), P (Pneumatic). Sensors: F (Force), P (Position), H (Pressure), C (Contact). Sealing: D (Drive), T (Tensioning), M (Mechanics), S (Sensors). Tested Depth: P (Pool), S (Sea), T (Tightness test only). Tested objects: O (Out of water), W (in Water).

**Gripper**	**Ref.**	**Year**	**Technology**	**#Fingers**	**#Joints**	**#Actuators**	**Weight**	**Dimensions**	**Finger Movement**	**Force/Torque/Speed**
TRIDENT-Skin	[53]	2013	Mixed	3	6	6	4.5 kg	L 300 mm W 250 mm	-	150 N per finger
PoseiDRONE	[58,59]	2013	Soft	1	-	1	-	LF 245 mm	-	-
TRIDENT-UNIBO	[54,55]	2014	Rigid	3	8	3	4.6 kg	LF 200 mm	Joints: 150°, 150°, 60°(abd.)	150 N per finger
ARTEMI	[60]	2014	Rigid	7	2	2	-	LF 450 mm	Roll: 360°, Open: 90°	Roll: 90°/s, Open: 20°/s
GUH14	[61]	2015	Rigid	3	9	3	-	-	Joint 1: 120°	-
UJIOne	[54]	2015	Rigid	4	2	1	3.93 kg	L 276 mm D 130 mm W 561 mm	-	-
MARIS	[56,57]	2017	Rigid	3	8	3	4.6 kg	-	Joints: 150°, 150°, 60°(abd.)	150 N per finger
IIT SoftHand	[17]	2018	Rigid	5	19	1	2 kg	L 170 mm Ø 95 mm	-	L 400 N, Pi 20 N, Po 76 N
IIT Gripper	[17]	2018	Rigid	4	-	1	2 kg	L 170 mm Ø 95 mm	-	Lift 150 N
OBSS	[62,63,64]	2019	Soft	4	n.a.	1	-	LF 100 mm	x: 145 mm, y: 110 mm	Pull off. 2 ÷ 10 N
Silver	[65]	2020	Soft	4	n.a.	1	-	L 125 mm Ø 48 mm	-	-
Tshingua	[66]	2020	Rigid	n.a.	1	1	-	-	90°	-
**Gripper**	**Actuation**					**Transmission**				**Sensors**
TRIDENT-Skin	E: Rotational motors					Worm gears				F, C: Optoelectronic
PoseiDRONE	E: Gear Motor GM12a Mini Metal					Tendons				-
TRIDENT-UNIBO	E: DC Brushless motor,12 W, Faulhaber					Worm gear, driving and joint pulleys, tendons routed around sheaths. Bicycle break-like mechanism (pretensioning)				F, C: Optoelectronic
ARTEMI	E: DC Geared motor					Gears				P: Optical encoders
GUH14	E: Servomotors, Hitec hs5646					Driving gear, belt, driven gears. Tendon (last two phalanges).				-
UJIOne	E: Servomotor Dynamixel AX-18F					Worm drive, spur gear				F: Strain gauge; C: FlexiForce.
MARIS	E: DC Brushless motor,12 W, Faulhaber					Worm gear (non-back-drivability) and tendons				F: Force/Torque (wrist)
IIT SoftHand	E: DC Gear motor, 12 V, Maxon DCX 22					Magnetic coupling, Gears and tendons				P: Magnetic encoders
IIT Gripper	E: DC Gear motor, 12 V, Maxon DCX 22					Magnetic coupling, Gears and tendons				P: Magnetic encoders
OBSS	P					Tubing				-
Silver	P					Tubing				-
Tshingua	E: Servomotor					Passive gears in pinion and rack set				-
**Gripper**	**Sealing Measures**					**Target**			**Test Depth**	**Tested Objects**
TRIDENT-Skin	M: Deformable silicon skin to be filled with incompressible oil mechanically pressed on the frame					Obj. Ø 5 ÷ 350 mm			P 5 m	W: Dummy black-box
PoseiDRONE	M: Silicone embedding					Wrapping around objects to hold them or to keep robot position			S 3 m	W: Cylinder, screwdriver
TRIDENT-UNIBO	D: Aluminum box with O-rings, PTFE-ring (shaft), Epoxy resin (supply and communication cable)					Obj. 5 ÷ 200 mm at 25 m depth, various grasp types			S 25 m	O: Bottle, card, pen, big box W: black box
ARTEMI	D: Watertight housing					Cylinders Ø 50 ÷ 500 mm			P 1.5 m	W: Thin pipe 1.5 kg, trash bin, T-bar handle
GUH14	O-rings (static) Bulkhead, double o-ring (dynamic)					Cylinders Ø 120 mm, Boxes 220 mm at 60 m depth			S 15 m, T	-
UJIOne	D: Cylindrical capsule; O-ring (connector); Lip ring seal (shaft). S: Self amalgamating tape.					Contingency plan while finishing the development of UNIBO			P	W: Dummy black-box
MARIS	D: Sealed independent capsule, O-rings (motor), PTFE-ring (shaft), Epoxy resin (supply and communication cable)					Obj. 5 ÷ 200 mm at 50 m depth, various grasp types, non-back-drivability			P	W: Cylinder Ø 10 cm
IIT SoftHand	D: watertight pressure-compensated chamber (electronics and motor)					Fine maintenance operations			S 10 m	O: Foam, metallic piece. W: Coin, vase shard, phantom coral and plant, valve.
IIT Gripper	D: watertight pressure-compensated chamber (electronics and motor)					Industrial diving scenario			S 10 m	O: Foam, metallic piece, paper tube, plastic sphere. W: Vase shards.
OBSS	Balancing Ambient/actuator pressure					Delicate grasping of sea organisms			S 10 m	O: Sphere 170 mm, beaker, cactus, CD, egg, milk bag, cylinders and cuboids. W: Sea urchins, cucumbers and shells
Silver	-					Delicate sample collection			S 1.2 m	W: Eggshell, plastic bottle, silicone seashell, plastic bag, finishing net
Tshingua	Waterproof cylinder base					Low-cost lightweight gripper for marine species			S	W: Sea urchins, cucumber and scallop

**Table 5:** Underwater gripper tested in the deep-sea with ROVs. Legend: # (Number of), n.a. (not applicable). Dimensions: L (Length), W (Width), D (Depth), LF (Finger length), Ø (Diameter). Force: P (Peak), NO (Normally operating). Actuation: H (Hydraulic), E (Electric), P (Pneumatic). Sensors: F (Force), P (Position), H (Pressure), C (Contact). Sealing: D (Drive), T (Tensioning), M (Mechanics), S (Sensors). Tested objects: O (Out of water), W (in Water), 2F, 3F, 4F or 5F (2-, 3-, 4- or 5-fingered grippers).

**Gripper**	**Ref.**	**Year**	**Technology**	**#Fingers**	**#Joints**	**#Actuators**	**Weight**	**Dimensions**	**Finger Movement**	**Force/Torque/Speed**
Wyss Boa	[10]	2016	Soft	1 + 2 (scissor)	1	1	-	LF 300 mm W 100 mm, D 110 mm	-	Pull 40 N Slip 30 N
Wyss Bellow	[10,67]	2016	Soft	4 + 2 (scissor)	1	1	-	LF 130 mm W 100 mm, D 110 mm	-	Pull 15 N Slip 5 N
Universal Jamming gripper	[71,72,73]	2016	Mixed	n.a.	n.a.	1	1.6 kg w/o particles	L 198 mm Ø 70 mm	-	18 ÷ 34 N
Ocean One	[45,74,75,76]	2017	Mixed	3	9	1	0.8 kg	L 94 mm W 150 mm D ~ 180 mm	Proximal Twist 20° Bend 110°; Medial: 120°	-
Wyss 3DP	[68]	2018	Soft	3	n.a.	1	-	-	-	-
Wyss Bellow v2	[67,68]	2018	Soft	2 (or 3 or 5)	n.a.	1	-	-	-	P: 16.6 N NO: 0,96 N
Wyss RAD	[69]	2018	Rigid	n.a.	~48	1	-	Max Ø 450 mm	n.a.	n.a.
JPL-Nautilus	[77]	2020	Rigid	16	32	1	-	L 750 mm	-	-
**Gripper**	**Actuation**				**Transmission**				**Sensors**	
Wyss Boa	H (fingers), Arm push-pull rod (scissor)				Valves (fingers), Bowden cable and 4 bar linkage (scissor)				-	
Wyss Bellow	H (fingers), Arm push-pull rod (scissor)				Solenoid Valves (fingers), Bowden cable and 4 bar linkage (scissor)				-	
Universal Jamming gripper	H				-				P: Pressure sensor referenced to ambient pressure	
Ocean One	E: brushless motor 70 W, Maxon EC-45; Return extension springs				Back-drivable gears, spring loaded winches that drives tendons				F: Pullout force estimated from suction flow	
Wyss 3DP	H				Tubing				-	
Wyss Bellow v2	H				-				-	
Wyss RAD	E: tilt motor, Saab Seaeye P00625				Passive revolute joints				-	
JPL-Nautilus	Handle impressed rotation				Lead screw, linear bearing, force balancing differential and tendons				-	
**Gripper**	**Sealing Measures**				**Target**		**Test Depth**		**Tested Objects**	
Wyss Boa	-				Quickly interchangeable soft gripper		170 m		O: Cylinders Ø 1 to 5 cm W: Whip coral	
Wyss Bellow	-				Quickly interchangeable soft gripper		170 m		O: Cylinders Ø 5 cm W: Soft coral, Scleratinia	
Universal Jamming gripper	O-rings (seal membrane and cap)				Universal compliant gripper		1200 m		O: Plastic comb, a hairbrush, a paint brush, and a two-pound dive weight. W: Allen key, weighted hairbrush, lightbulb, metal spring, lightbulb, wine glass, shell, weighted GoPro housing, plastic safety glasses, clam shell	
Ocean One	D: Pressure-compensated oil-filled chamber				Substitute human divers		91 m		O: Battery, goblet, screwdriver, plate, Wood block, PVC Tube, Plastic pear, Mug, W: Rope, archeology tools, crate amphora	
Wyss 3DP	3000 m 1 atm housing with external power				-		1950 m		W: Crinoid, sponge, coral, sea star, cucumber	
Wyss Bellow v2	3000 m 1 atm housing with external power				Obj. up to Ø 140 mm		2440 m		W: 5F: Holoturia 4F: Coral rubble 3F: Coral rubble, sponge, Fossil shelves, Pyrosome, Cirrothauma murrayi 2F: Holoturia, Hexatinellid sponge	
Wyss RAD	D: oil-filled pressure compensated unit				Envelope delicate specimens		645 m		W: Oegopsina sp. Squid, Stellamedusa ventana, Stigmatoteuthis sp. Squid	
JPL-Nautilus	-				Grip curved surface with asperities		2000 m		O: spheres from 4 to 33 diameter, rocks, seashell, asperity grasping test W: loose rocks and asperity grasping test	

**Table 6:** Underwater grippers and tools offering a specific action possibility. Actions in black were taken from [15]. Grippers analyzed in this review were tested for additional actions, which are reported here in red. For commonly used grippers: ✓ Possible; T Possible with tool; T* Potentially possible, provided that the gripper can handle the tool; x Not possible. For tools: T Tool used as tool; M Tool used as manipulated object. For research status gripper, the following symbols add to the previous ones: ✓* The design suggests the possibility of action but there is no test yet; x* the design suggests impossibility of action but there is no test yet. 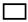 White spaces mean that we cannot state a priori whether the action is possible; 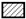 patterned spaces mean that we cannot state a priori whether the gripper has the ability to handle the tool. 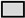 Shaded spaces represent not applicable cases.

Atomic Manipulations	Push to Break	Scrape	Scrape with Tool	Scoop	Scoop with Tool	Core with Corer	Grip	Grip and Twist	Grip and Pull	Grip Tool	Cage	Cage and Pull	Suction on with tool	Suction and Store	Suction off with tool	Release	Release Tool	Pour	Pour with Tool	Suction on	Suction off	Scissor Cut	Lever	Pull (Hook)
Gripper or tool	1.1	2	2.A	3	3.A	4.A	5	5.1	5.2	5.B	6	6.1	7.A	8.A	9.A	10	10.B	11	11.A	7.	9.	12.	13.	14.
Parallel fingers	✓	✓	T	x	T	T	✓	✓	✓	✓	x	x	T	T	T	✓	✓	x	✓	x	x	x	✓	✓
Intermeshed fingers	✓	✓	T	x	T	T	✓	✓	✓	✓	x	x	T	T	T	✓	✓	x	✓	x	x	x	✓	✓
Grabber	✓	✓	T	✓	T	T	x	x	x	x	✓	✓	T	T	T	✓	✓	✓	✓	x	x	x	x	x
Cage	x	x	T*	x	T*	T*	x	x	x	x	✓	✓	T*	T*	T*	✓	✓	x	x	x	x	x	x	x
Suction Sampler			T										T	T	T									
Push corer						T											M							
Baskets			T		T												M		T					
Scoop			T		T														T					
Scoop net			T		T												M		T					
Trap										M							M							
AMADEUS			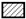	x	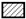	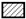	✓			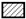	x	x	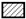	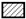	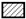	✓	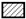	x	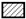	x	x	x		
HEU II			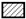	x	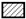	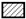	✓			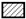	x	x	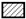	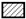	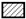	✓	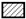	x	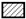	x	x	x		
SeeGrip			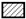	x	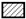	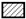	✓			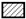	x	x	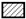	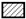	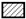	✓	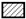	x	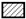	x	x	x		
Okinawa	✓*	✓*	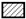	x	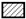	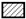	✓			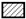	x	x	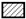	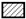	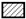	✓	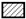	x	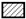	x	x	x	✓*	✓*
Stanford	x	x	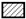	x	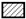	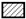	✓			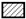	x	x	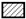	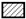	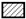	✓	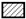	x	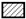	✓	✓	x	x	
Malaysia Pahang	✓*	✓*	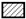	x	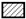	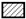	✓			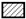	x	x	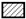	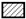	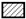	✓	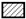	x	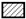	x	x	x	✓*	✓*
Calabria	x	x	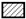	x	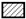	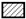	✓			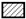	x	x	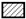	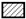	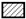	✓	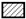	x	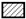	x	x	x	x	
Tokai			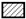	x	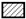	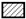	✓			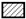	x	x	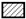	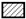	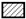	✓	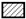	x	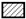	x	x	x		
Wyss Ultragentle	x	x	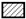	x	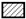	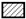		x*		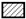	✓		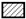	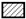	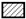	✓	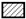	x	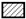	x	x	x	x	✓
VSPP-3	x	x	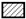	x	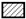	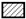	✓			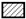	x	x	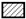	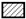	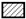	✓	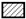	x	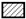	x	x	x	x	
TRIDENT-Skin	x	x	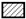	x	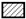	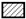	✓			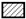	x	x	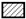	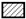	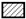	✓	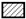	x	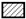	x	x	x	x	
PoseiDRONE	x	x	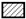	x	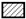	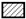	✓	x*		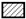	x	x	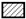	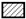	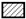	✓	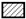	x	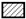	x	x	x	x	
TRIDENT-UNIBO	x	x	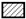	x	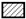	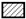	✓			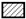	x	x	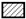	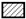	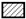	✓	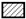	x	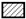	x	x	x	x	
ARTEMI	✓*	✓*	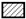	x	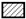	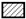	✓	✓		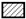	x	x	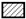	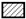	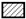	✓	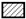	x	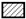	x	x	x	✓*	✓*
GUH14	x	x	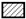	x	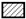	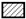	✓*			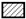	x	x	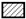	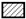	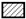	✓*	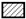	x	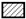	x	x	x	x	
UJIOne			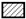	x	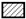	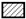	✓			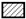	x	x	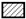	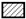	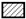	✓	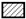	x	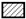	x	x	x		✓
MARIS	x	x	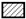	x	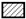	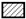	✓			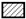	x	x	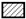	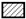	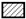	✓	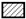	x	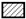	x	x	x	x	
IIT SoftHand	x	x	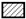	x	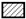	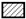	✓	✓		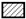	x	x	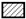	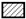	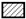	✓	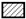	x	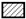	x	x	x	x	x
IIT Gripper	x	x	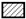	x	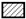	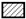	✓			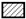	x	x	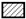	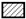	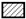	✓	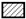	x	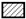	x	x	x	x	x
OBSS	x	x	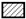	x	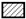	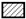	✓*	x*		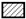	✓		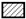	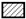	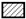	✓	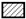	x	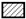	x	x	x	x	
Silver	x	x	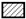	x	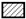	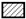	✓	x*	✓	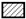	✓	✓*	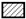	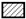	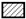	✓	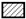	x	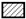	x	x	x	x	
Tshingua	x	x	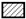	x	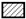	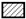	x	x	x	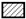	✓		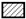	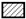	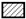	✓	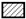	x	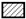	x	x	x	x	x
Wyss Boa	x	x	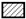	x	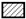	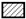	✓	x*	✓	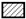	x	x	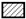	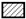	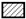	✓	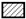	x	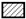	x	x	✓	x	
Wyss Bellow	x	x	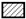	x	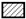	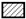	✓			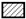	x	x	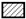	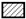	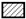	✓	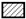	x	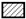	x	x	✓	x	
Universal Jamming	x	x	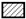	x	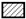	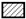	✓		✓	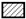	✓	✓	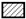	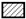	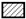	✓	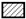	x	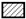	x	x	x	x	x
OceanOne	x	x	✓	x	✓	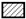	✓			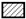	x	x	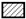	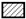	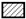	✓	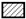	x	✓	✓*	✓*	x	x	
Wyss 3DP	x	x	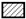	x	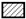	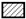	✓	x*		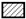	✓		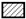	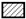	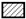	✓	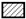	x	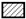	x	x	x	x	
Wyss Bellow v2	x	x	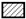	x	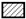	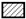	✓	x*		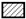	✓		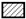	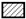	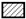	✓	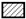	x	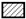	x	x	x	x	
Wyss RAD	x	x	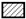	x	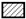	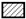	x	x	x	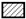	✓	x	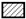	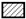	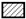	✓	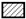	x	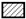	x	x	x	x	x
JPL-Nautilus	x	x	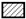	x	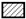	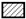	✓			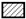	✓		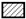	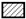	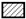	✓	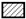	x	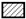	x	x	x	x	✓

## Data Availability

Data is contained within the article.
